# Preliminary Assessment of *Arnica montana* L. Extract: Antimicrobial Activity Against *Acinetobacter baumannii* and Biofilm-Related Gene Expression Profiling

**DOI:** 10.3390/genes16121473

**Published:** 2025-12-09

**Authors:** Sylwia Andrzejczuk, Magdalena Sozoniuk, Danuta Sugier

**Affiliations:** 1 Department of Pharmaceutical Microbiology, Medical University of Lublin, Chodźki 1, 20-093 Lublin, Poland; 2 Institute of Plant Genetics, Breeding and Biotechnology, University of Life Sciences in Lublin, Akademicka 15 St., 20-950 Lublin, Poland; 3 Department of Industrial and Medicinal Plants, University of Life Sciences in Lublin, Akademicka 15 St., 20-950 Lublin, Poland; danuta.sugier@up.edu.pl

**Keywords:** *Arnica montana* L., antibacterial activity, sub-inhibitory MIC, biofilm, gene expression

## Abstract

**Background/Objectives**: *Arnica montana* L. is widely recognized for its diverse biological activities, including antimicrobial effects. This study aimed to evaluate the antimicrobial and antibiofilm activity of *A. montana* L. extracts against *Acinetobacter baumannii*, a pathogen of urgent public health concern due to its increasing antibiotic resistance and capacity for biofilm formation. **Methods**: The antimicrobial activity of ethanolic (EtE) and aqueous (AqE) extracts of *A. montana* flowers was evaluated via the broth microdilution method. The minimal inhibitory concentration (MIC) and minimal bactericidal concentration (MBC), and the MBC/MIC ratio were used. The effects of EtE on *A. baumannii* biofilm formation were assessed via a crystal violet assay. Additionally, transcriptional profiling of biofilm-associated genes following exposure to sub-MIC levels of the extract was conducted via RT-qPCR. **Results**: The anti-*Acinetobacter* activity of EtE was demonstrated (MIC = 234.4 and 468.75 µg/mL for *A. baumannii* ATCC BAA-3252 and ATCC 19606, respectively). The EtE exhibited bactericidal activity against both strains, whereas the AqE showed no activity. Additionally, EtE inhibited biofilm formation and significantly downregulated the expression of key biofilm-associated genes, including those of the *csu* operon and *ompA*. **Conclusions**: *Arnica montana* EtE demonstrated antimicrobial and antibiofilm activities against *A. baumannii* and inhibited biofilm development by suppressing the transcription of genes involved in pilus assembly and surface adherence, highlighting their essential role in biofilm formation.

## 1. Introduction

The present-day global problem of drug resistance among bacteria and fungi is escalating in severity. The use of plant-based products and natural extracts has emerged as a promising alternative to pharmacological interventions in the fight against multidrug-resistant (MDR) pathogens. In contemporary practice, their potential is most frequently utilized as antibiotic “enhancers” and/or biofilm inhibitors, with less frequent use as standalone drugs. Many secondary metabolites produced by plants provide chemical defenses against a wide range of infectious agents, including pathogenic microorganisms. The rich diversity of these phytochemicals offers valuable resources for developing new antimicrobial agents to combat MDR-ESKAPE pathogens such as *Enterococcus* spp., *Staphylococcus aureus*, *Klebsiella pneumoniae*, *Acinetobacter baumannii*, *Pseudomonas aeruginosa*, and *Enterobacter* spp. [1]. A number of plant extracts and plant-derived compounds have been shown to possess antimicrobial properties. For instance, myristic acid (a fairly common form of saturated fatty acid) has been found to be effective against *S. aureus* [2]; quercetin-7-O-methyl ether and isoorientin, isolated from *Rhynchosia beddomei* Baker, have shown antibacterial properties against *P. aeruginosa* and *S. aureus* [3]; and Song et al. [4] reported that isopentenylated flavonoids (α-mangostin and isobavachalkone) were effective against antibiotic-resistant *E. faecium* and *S. aureus*. In their seminal study, Idris et al. [1] comprehensively summarized the extant literature on the subject, meticulously collecting data from numerous publications detailing the activity of various plant extracts against ESKAPE pathogens. The data presented by the authors revealed a paucity of phytochemicals with high potential for inhibiting the growth or killing of *A. baumannii* cells. However, an exception to this is observed with the ethanol extract from *Lavandula stoechas* flowers, for which MIC against planktonic bacteria of 35.9 μg/mL has been reported [5]. To date, several antimicrobial mechanisms of action of plant components have been described. Plant bioactive compounds have been demonstrated to function as inhibitors of efflux pumps (for instance, baikalin, catechins, and piperine) [6]. Natural compounds can act by modifying target proteins of selected pathogens, as exemplified by sulfur compounds from garlic (allicin/ajoene) and isothiocyanates (e.g., benzyl-ITC) [7].

As posited by numerous scientists, a further mechanism involves membrane disruption and osmotic stress (in the context of essential oils), with thymol, carvacrol, eugenol, cinnamaldehyde, and terpinen-4-ol being notable examples [8,9,10,11]. These substances have been demonstrated to compromise the integrity of bacterial and fungal cell membranes, thereby diminishing their capacity to establish biofilms and impeding the process of quorum sensing. In numerous models, these compounds are combined in vitro with antibiotics (e.g., amikacin), which results in a substantial reduction in the MIC against MDR bacteria of the genera *Klebsiella*, *Acinetobacter*, and *Staphylococcus* [10]. Compounds of natural origin have also been shown to possess antibacterial and/or antiadhesive properties, thereby impeding the adhesion of microorganisms to both biotic and abiotic surfaces [12]. Essential oils (e.g., cinnamon, clove, and lemongrass) and polyphenolic compounds (e.g., quercetin, kaempferol-3-rutinoside, and galangin) [12,13,14] have been shown to reduce various bacterial cell processes. For example, the processes of biofilm formation and maturation of *Vibrio parahaemolyticus,* which carry virulence-associated genes, are effectively inhibited at sub-MICs by lemongrass and cumin essential oils [13].

*Arnica montana* is a medicinal plant widely recognized for its antimicrobial, anti-inflammatory, antitumor, and analgesic properties. The multiple pharmacological applications of *A. montana* are attributed to its diverse bioactive compounds, including sesquiterpene lactones (e.g., helenalin, 11α,13-dihydrohelenalin), sesquiterpenes (e.g., caryophyllene, germacrene D), and monoterpenes (e.g., thymol, α-pinene), as well as eugenol, flavonoid glycosides, fatty acids, and oligofructozides, all of which collectively contribute to its broad therapeutic potential [15,16,17]. According to Thakur [16] and other researchers [18], arnica supports various functions in the body, including improving blood circulation, reducing muscle pain and swelling, and accelerating healing after surgery or injury. Arnica extracts have also demonstrated promising antibacterial activity against several pathogenic microorganisms [19,20,21,22], making them potential candidates for developing alternative prophylactic and novel antimicrobial therapies. However, the extant literature contains a paucity of studies that have examined the effects of *A. montana* extracts on *Acinetobacter* spp.

*Acinetobacter baumannii* is a Gram-negative, aerobic, opportunistic pathogen that poses a significant threat to patients with compromised immune systems (who experience prolonged hospitalization of more than 90 days). It is also a primary cause of various infections, including pneumonia (ventilator-associated pneumonia, community-acquired pneumonia), urinary tract infections, bloodstream infections (e.g., bacteremia, sepsis), nosocomial postneurosurgical meningitis, and wound and soft tissue infections [23,24]. As stated by many authors [23,25], this bacterium naturally inhabits water and soil environments, as well as food or medical devices. The pathogenesis is strongly attributed to a variety of virulence factors, including adhesins, lipooligosaccharides, outer membrane proteins, iron-chelating systems, protein secretion systems, and enzymes (phospholipases, proteases), among others [24,25]. One of the most significant factors contributing to the pathogenicity of *A. baumannii* is the ability of the bacterium to adhere to, form biofilms on, and colonize both biotic and abiotic surfaces, with a prevalence ranging from 32 to over 90% and simultaneously carrying multiple biofilm-related genes [24,25,26].

The primary threat posed by the presence of *A. baumannii* in the environment and human surroundings is the continuous development of antibiotic resistance, which is particularly alarming in the case of frequently used carbapenems (carbapenem-resistant *A. baumannii*, CRAB) and can lead to infections that are difficult to treat [24]. Consequently, *A. baumannii* is designated the fourth most critical pathogen by the World Health Organization in the critical group category. A significant constraint in efforts to combat these pathogens is the paucity of promising pharmaceutical agents currently under development. Furthermore, infections caused by critical pathogens can also be extremely difficult to prevent and are highly contagious [23,27]. Several comprehensive reviews have provided detailed insights into the mechanisms and stages of biofilm development in *A. baumannii* [25,26,28]. The inhibition of biofilm formation represents a promising therapeutic approach for combating *A. baumannii* infections, as biofilms significantly contribute to pathogen resistance to antibiotics and persistence in clinical environments. Targeting biofilm development can disrupt bacterial colonization and increase the efficacy of antimicrobial treatments.

This preliminary study evaluated the antibacterial and antibiofilm effects of *A. montana* flower extract (both aqueous and ethanolic) against two reference strains of *A. baumannii,* either planktonic or biofilm-forming cells, with a particular focus on transcriptional analysis of selected biofilm-associated genes to elucidate the molecular responses induced by the treatment and to provide insights into its antibiofilm activity.

## 2. Materials and Methods

### 2.1. Plant Material and Extraction Procedure

The plant material, consisting of *Arnica montana* L. flower heads in the full flowering phase, was collected from the experimental field of the University of Life Sciences in Lublin, eastern Poland (51°31′39.5″ N, 22°45′6.7″ E). After harvesting, the flower heads were air-dried at 40 °C, finely ground using a laboratory mill, and subsequently sieved (passing through a nylon sieve with a mesh size of 1 mm) to obtain homogeneous powdered plant material for further analyses. Five-gram portions of raw material were weighed and transferred into 250 mL Erlenmeyer flasks. Each sample was then supplemented with 50 mL of the appropriate solvent: either distilled water or 70% (*v*/*v*) ethanol. The flasks were tightly sealed and placed in a shaking water bath at 40 °C for 5 h. Upon completion of the extraction, the solutions were filtered through a plastic Büchner funnel fitted with cellulose filter paper. The resulting extracts were subjected to further analyses, while the plant material remaining after extraction was discarded.

### 2.2. Chemical Analyses

The content of sesquiterpene lactones was quantified via the chromatographic method described in the Polish Pharmacopoeia IX [29]. The results are expressed as a percentage of the total sesquiterpene lactone content, which was calculated as dihydrohelenalin tiglinate equivalents. The flavonoid content, expressed as quercetin equivalents, was determined via the spectrophotometric method specified in the Polish Pharmacopoeia VIII [30]. Each marking was performed in four replicates. Representative data are shown.

### 2.3. Microorganisms

Two reference strains, *Acinetobacter baumannii* ATCC 19606 and *A. baumannii* ATCC BAA-3252, obtained from the American Type Culture Collection (ATCC), were used. Bacteria were kept frozen at −70 ± 2 °C in brain heart infusion broth (BHI, Oxoid, Milan, Italy). They were cultured for 24 h at 35 ± 2 °C in Tryptic Soy Agar (TSA, Oxoid, UK) and then used to test the antimicrobial potential of the extracts.

### 2.4. Antimicrobial Activity Test

The objective of this study was to determine the antibacterial potential of ethanolic and aqueous extracts of *Arnica montana* L. against *Acinetobacter* spp. bacteria. The broth double microdilution method was utilized, as stipulated by the European Committee on Antimicrobial Susceptibility Testing (EUCAST), as the reference method for determining the antimicrobial potential of natural products and newly synthesized compounds, as well as compounds of an antibiotic and chemotherapeutic nature [31]. The quantitative activity of the tested products was measured with the following parameters: the minimum inhibitory concentration (MIC), defined as the lowest concentration of product tested that inhibits the in vitro visible growth of microorganisms expressed in μg/mL; the minimum bactericidal concentration (MBC), defined as the lowest concentration of product tested that results in the absence of visible growth of microorganisms, expressed in μg/mL. Furthermore, the MBC:MIC ratios were also calculated to evaluate the nature of the formulations’ activity. The product was classified as bactericidal if the MBC:MIC values were ≤4 and as bacteriostatic if the MBC:MIC values were in the range of 4–32 [21,32].

The method involved the preparation of microbial suspensions at a standardized density (0.5 on the McFarland, McF) standard scale, corresponding to 1.5 × 10^8^ CFU/mL and an optical density of OD_550_ = 0.125. The assay was performed within the concentration range of 7.32–30,000 µg/mL on polystyrene 96-well microplates (Medlab, Raszyn, Poland), with 200 µL of the initial test product concentration and 100 µL of sterile microbiological Mueller-Hinton broth medium (MHB, Biomaxima, Lublin, Poland) added to each well, as previously described [33]. Concurrently, the following controls were conducted: (a) a medium control consisting of 100 µL of sterile MHB medium devoid of microorganisms, (b) a microbial viability control comprising 100 µL of sterile medium with 2 µL of microbial suspension lacking extracts tested, (c) the *A. montana* extracts control involving serial twofold dilutions without added microorganisms (conducted in the same concentration ranges as the actual experiment; the OD_600__values read in these wells were used as a reference point for determining the MIC), and (d) amoxicillin (5 mg/mL, Glentham Life Sciences, Corsham, UK), as well as 70% ethanolic alcohol as a control of the solvent (to strictly adhere to the experimental protocol, the control conditions accurately reflected the procedure used for the bacterial strains under investigation, except for the addition of pure solvent in the same volumes as the extract. In this way, a series of double ethanol dilutions ranging from 0.03 to 70.0% was obtained, corresponding to dilutions of the tested extracts ranging from 7.32 to 30,000 µg/mL). The microtiter plates were then subjected to an incubation period of 18 ± 2 h at 35 ± 2 °C. A spectrophotometric assay was subsequently conducted on each well of the plate, with measurements recorded at 600 nm via an ELx800 plate reader (BioTek Instruments, Winooski, VT, USA). The MIC values were then determined in those wells where the extracts studied completely inhibited the growth of the reference microbial strains. The MBC values were determined by plating 5 µL of the contents of each well of the microtiter plates showing growth inhibition on the Mueller-Hinton agar (MHA, Biomaxima, Lublin, Poland) plates. The area on the plate where 99.9% inhibition of microbial growth (planktonic cells) was observed was identified as the lethal concentration. The experiment was performed in at least triplicate, and representative data are shown.

### 2.5. Anti-Biofilm Assay

The biofilm-forming ability of the tested *Acinetobacter* spp. strains and the antibiofilm potential of the ethanolic extract of *Arnica montana* L. were measured quantitatively via a modified 0.1% crystal violet (CV, Oxoid, UK) method based on the OD_570_ [34]. The biofilm assay was performed on Nunc™ MicroWell™ 96-well, Nunclon Delta-Treated, flat-bottom microplates (Thermo Fisher Scientific, Waltham, MA, USA). Bacterial suspensions were prepared in Tryptic Soy Agar (TSA, Oxoid, Basinstoke, UK) at a 0.5 McFarland density. A quantity of 200 μL of each arnica extract was added to the first wells in a row and then diluted as previously described to achieve the same dual dilutions. In the final stage, 2 µL of bacterial suspension (0.5 McF) was added to each well and then incubated for 24 h at 37 °C. The medium above the culture was then decanted. The microplates were meticulously washed multiple times with distilled water to remove nonadherent or loosely adherent cells. Thereafter, they were dried in an inverted position and stained with 200 μL of 0.1% CV. The plates were left to stand for 15 min, after which the dye was removed, and each well was rinsed several times with sterile distilled water to ensure the removal of nonadherent forms. Finally, the microtiter plates were dried, filled with 200 μL of 96% ethanol (POCH, Lublin, Poland), and the optical density (OD_570_) was measured spectrophotometrically at a wavelength of 570 nm via an ELx 800 microplate reader (BioTek Instruments, Winooski, VT, USA) after 15 min [35]. Based on absorbance values, both *Acinetobacter* spp. strains were classified as biofilm-forming producers as described previously [36]. Analogue types of control were used: MHB control medium without the tested extract; control medium with extract added, but no bacterial inoculum added, and a series of dilutions (negative control). The OD_570_ values read from these wells were used as reference points to determine the minimal biofilm inhibitory concentration (MBIC) values. The viability of the tested strains, representing 100% growth without the addition of the tested extract, was checked independently. The experiment was performed in triplicate, and representative data are shown.

Based on the obtained data, the MBIC of the tested extract that inhibits biofilm formation by *A. baumannii* was determined. This value was determined in wells containing the lowest concentration of the extract (the highest dilution), in which a significant reduction or inhibition of biofilm formation compared to the untreated control was observed. The percentage of biofilm inhibition can be calculated using the formula [(OD_control_ − OD_sample_)/OD_control_] × 100 [37], where OD_control_ was the absorbance of the positive control wells (untreated bacteria), and OD_sample_ was the absorbance of the wells treated with the extract. Biofilm inhibition was rated on a scale of 0–100%; values <0% indicated biofilm growth enhancement, while values between 0 and 50% showed weak anti-biofilm activity; values >50% represented good biofilm inhibition. All image analysis was performed using the ImageJ 1.54g software (US National Institutes of Health, Bethesda, MA, USA) to calculate the measured area occupied by biofilm (MAOB).

### 2.6. RNA Extraction and cDNA Synthesis

To assess transcriptional changes in genes associated with biofilm formation in *A. baumannii*, treatments included subinhibitory (sublethal) MIC concentrations: ½ MIC and ¼ MIC of *A. montana* extract, ethanol at equivalent concentrations (½ MIC and ¼ MIC), and an untreated control. The RNA extraction was performed in accordance with the manufacturer’s instructions using TRIzol reagent (Invitrogen, Carlsbad, CA, USA). Immediately following isolation, the RiboLock RNase Inhibitor 40 U/μL (Thermo Scientific, Waltham, MA, USA) was added to the samples. The extracted RNA was then assessed spectrophotometrically with a NanoDrop2000 (Thermo Scientific, Waltham, MA, USA) and electrophoretically on a 1.5% agarose gel stained with ethidium bromide. The potential genomic DNA contamination was removed using RNase-free DNase I (EURx, Gdańsk, Poland) following the manufacturer’s recommendations. The reverse transcription reactions were carried out using the NG dART RT Kit (EURx, Gdańsk, Poland) on 1.5 µg of RNA. The resulting cDNA was diluted and used as the template for subsequent RT-qPCR reactions.

### 2.7. RT-qPCR

Following a comprehensive review of the extant literature, six genes of interest *(bap*, *csuA*, *csuB*, *csuC*, *csuD*, and *ompA*) were selected for expression analysis via RT-qPCR. Both bacterial strains were tested for the presence of genes via classical PCR, visualized on a 1.5% agarose gel during electrophoresis, photographed using a Quantum ST4 system (Vilber Lourmat, France), documented, and archived via Quantum ST5 Xpress v 16.08g computer software (Vilber Lourmat, Marne-la-Vallée, France).

**Figure A1:**
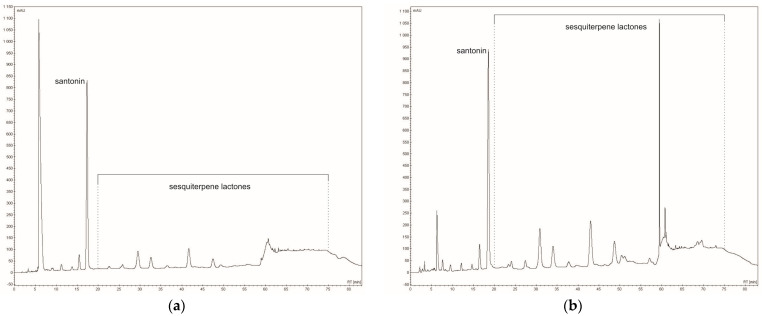
The HPLC at 225 nm chromatogram of sesquiterpene lactones in (**a**) aquatic and (**b**) ethanolic extracts of *Arnica montana* L.

The primer sequences for the GOIs were designed using the PrimerBLAST tool [38]. The primers used for reference genes (*rpoB* and *rpoD*) and the *bap* gene were obtained from the literature [39,40]. The primer specifications are outlined in Table 1. The amplification specificity was then assessed by melting curve analysis. All primer pairs used in this study exhibited specific amplification, as confirmed by the presence of single peaks on the generated dissociation curves (Figure A1). The amplification efficiency was determined via analysis of standard curves prepared from serial dilutions of pooled cDNA. The RT-qPCR reactions were performed using SG/ROX on*Taq* qPCR Master Mix (2×) (EURx, Gdańsk, Poland) on 50 ng of cDNA with optimized primer concentrations (Table 1). The thermal cycling conditions were as recommended by the supplier (15 min at 95 °C for initial denaturation, followed by 40 cycles of 15 s at 94 °C, 30 s at 60 °C, and 30 s at 72 °C, followed by a dissociation curve step with continuous data collection from 60 °C to 95 °C). All reactions were carried out in three technical replicates along with no-template controls (NTCs) on a QuantStudio™ 6 Pro Real-Time PCR System (Applied Biosystems™, Thermo Fisher Scientific, Waltham, MA, USA). The relative expression levels of the target genes (2^−∆∆Ct^ method) were calculated using the relative quantification module in Thermo Fisher Cloud (Thermo Fisher Scientific, Waltham, MA, USA).

### 2.8. Statistical Analysis

Statistical analyses were performed to assess the significance of differences between the experimental groups. Homogeneity of variances was tested using Levene’s test prior to parametric analyses. For parametric data with equal variances, one-way analysis of variance (ANOVA) followed by Tukey’s post hoc test was used for multiple comparisons. For nonparametric data, the Mann–Whitney U test was applied to compare differences between groups. The results were considered statistically significant at a significance level of *p* < 0.05. The experimental data are shown as the means ± standard deviations (SDs). The statistical tests were performed via Statistica 13.1 software (StatSoft, Inc., Tulsa, OK, USA), and the graphs were created using GraphPad Prism 8 version 8.4.2 (GraphPad Software, LLC, San Diego, CA, USA).

## 3. Results

### 3.1. Chemical Analysis of Extracts

The ethanolic extract of *A. montana* flowers contained approximately 2.5-fold higher concentrations of sesquiterpene lactones (STLs) compared to the aqueous extract (1.645% versus 0.670%, respectively). Furthermore, the flavonoid content was also greater in the ethanolic extract, reaching 0.725%, while in the aqueous extract, it amounted to 0.680%. Figure A1 in Appendix A presents an example HPLC chromatogram of STLs in aquatic (A) and ethanolic (B) extracts.

### 3.2. Antibacterial Activity Assay Against A. baumannii Planktonic Cells

The findings outlined in Table 2 were derived from experimental trials employing the broth double dilution method. The MIC values for the planktonic cells of the reference strains of *A. baumannii* in the presence of the ethanolic extract ranged from 234.4 to 468.75 µg/mL, while the MBC values were the same for both strains at 937.5 µg/mL (Table 2). The alcoholic extract from arnica flowers demonstrated bactericidal properties against both *Acinetobacter* spp. reference strains, with MBC/MIC ratios of 2 and 4, for *A. baumannii* ATCC 19606 and ATCC BAA-3252, respectively. Conversely, the aquatic extract did not present any potential activity against the strains studied. The MIC values exceeded the concentration range used.

**Figure 1:**
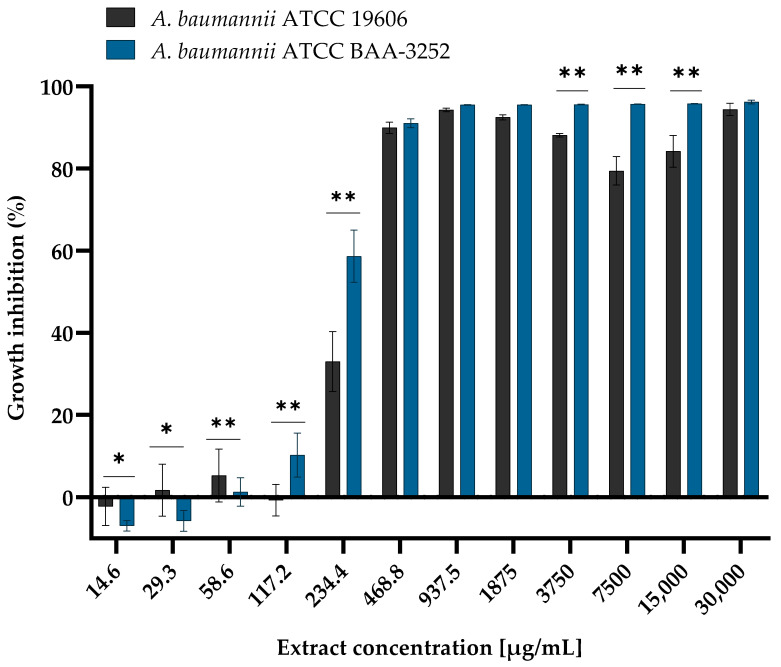
Reduction in planktonic cells growth of *Acinetobacter baumannii* reference strains ATCC 19606 and ATCC BAA-3252 treated with different concentrations of the *Arnica montana* L. ethanolic extract after 24 h of incubation at a temperature of 37 °C in aerobic conditions. Explanatory notes: mean OD_600_ ± SD values for both strains control without extract (100% growth of the non-treated bacterial strains) were 1.0206 ± 0.019 and 1.0295 ± 0.028 for *A. baumannii* ATCC 19606 and *A. baumannii* ATCC BAA-3252. Statistical data were analyzed using the Mann–Whitney U nonparametric test (with continuity correction). The results marked with an asterisk (*) are statistically significant at *p* < 0.05; (**) are significant at *p* < 0.01. Data were based on ten replicates for each bacterial strain.

The MIC values showed that arnica ethanol extract was active against planktonic cells of the reference strains of the species *A. baumannii*. The multiplication of both strains of *A. baumannii* increased in the same way when the extract was present (Figure 1). They were gradually inhibited, and the extent of this inhibition was dependent on the concentration of the ethanolic extract. At low concentrations ranging from 14.6 to 58.6 µg/mL, bacterial growth was comparable to that of the growth control (the strain not exposed to the extract, 100% growth). The OD_600_ values for *A. baumannii* ATCC 19606 ranged from 1.032 ± 0.040 to 0.962 ± 0.063, and for *A. baumannii* ATCC BAA-3252 ranged from 1.019 ± 0.016 to 0.943 ± 0.038 (Table A1). There was a important decrease in bacterial proliferation just before a very high concentration of the extract (between 234.4 and 468.8 µg/mL). An inhibition of planktonic cells’ growth of both strains was reached at those concentrations of extract, the levels between 34.2–89.9 ± 0.007% for *A. baumannii* ATCC 19606, and between 69.6–93.1 ± 0.020% for ATCC 3252 (difference between strains was statistically significant with *p* < 0.05; Figure 1). A strong reduction in bacterial growth was observed at MIC values exceeding the ethanolic extract concentration of 468.8 µg/mL. The growth of planktonic cells was inhibited in both *Acinetobacter* spp. strains, with an estimated mean level of 88.8–92.4 ± 0.0140 for ATCC 19606 strain and 89.7–92.4 ± 0.0109% for ATCC BAA-3252 strain (*p* = 0.999). Statistically significant differences were observed at sublethal concentrations (*p* < 0.0001). Moderate inhibition of the growth of planktonic cells of these bacteria was caused by the presence of >468.8 μg/mL of the extract, with growth inhibition ranging from 87.4 ± 0.0046% to 96.6 ± 0.0136% for *A. baumannii* ATCC 19606 and from 95.4 ± 0.0005% to 96.9 ± 0.0042% for *A. baumannii* ATCC BAA-3252 (statistically significant difference was noted at every extract concentrations except 30,000 µg/mL; *p* < 0.0001).

It is important to note that the MIC values for pure ethanol were 2-fold higher than the MIC values (937.5 and 1875.0 µg/mL for *A. baumannii* ATCC 19606 and ATCC 3252, respectively). Furthermore, the growth of bacterial planktonic cells was also caused by the presence of ethanol (at concentrations corresponding to an MIC of >468.8 μg/mL of the extract). Growth inhibition ranged from 89.7 ± 0.013% to 96.9 ± 0.004% for *A. baumannii* ATCC 19606, and from 81.9 ± 0.045% to 95.3 ± 0.003% for *A. baumannii* ATCC 3252. A statistically significant difference was observed between ethanol-treated and non-treated cells of both strains (*p* < 0.0001).

### 3.3. Effects of Arnica Ethanolic Extract on A. baumannii Biofilm Cells

**Figure 2:**
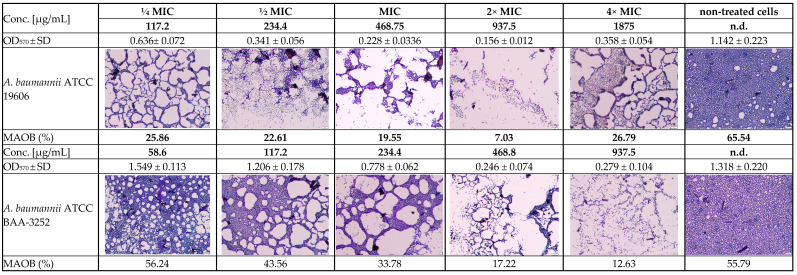
Light inverted microscopy observation of the subinhibitory MIC antibiofilm effects of the *Arnica montana* ethanolic extract on *Acinetobacter baumannii* reference strains (ATCC 19606 and ATCC BAA-3252 under stationary conditions (2000× magnification, 20 µm scale bar, Olympus DP22 automated inverted light microscope, CellSens Dimensions 2.3 software, Olympus Corporation, Tokio, Japan). Explanatory notes: OD_570_ ± SD—median values of OD_570_ of four replicates with standard deviation; MAOB—measured area occupied by biofilm (%) calculated via ImageJ 1.54g software (US National Institutes of Health, Bethesda, Maryland, USA); strain control without extract means the 100% growth of the non-treated bacterial strain.

The arnica extract (initial concentration of 30,000 µg/mL) was tested on biofilm cells at twelve different concentrations (corresponding to dilutions at the antimicrobial assay), including subinhibitory concentrations of ½ and ¼ MIC, as well as 2- and 4-fold the respective planktonic MIC (2*×* and 4*×* MIC), which significantly inhibited biofilm formation across both reference *A. baumannii* ATCC 19606 and ATCC BAA-3252 strains (Figure 2). The presence of arnica ethanol extract in the culture medium had a similar effect on the biofilm formation of *A. baumannii* strains ATCC 19606 and ATCC BAA-3252.

**Figure 3:**
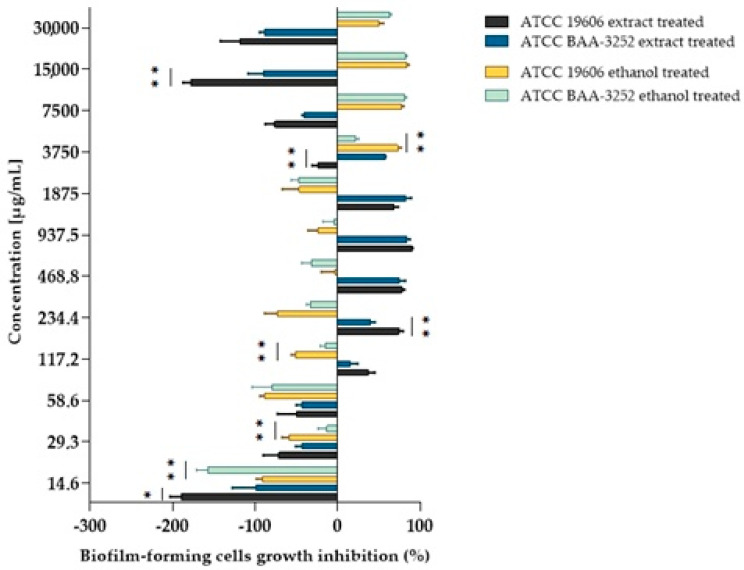
The inhibition of biofilm-forming cell growth depends on the *Acinetobacter baumannii* strain and the extract and/or alcohol used. Explanatory notes: biofilm inhibition rated on a scale of 0–100%; values < 0% indicated bacterial biofilm growth enhancement; values of 0–50% indicated weak anti-biofilm activity; values > 50% indicated good biofilm inhibition; Statistical data were analyzed using the Mann–Whitney U test between bacterial strains treated with plant extract or between bacterial strains treated with ethanol. The asterisks mean statistically significant differences as follows: (*) are statistically significant at *p* < 0.05; (**) are significant at *p* < 0.01. Data were based on four replicates.

The ethanolic Arnica extract had various levels of biofilm inhibitory activity against both *Acinetobacter* spp. strains. The extracts showed more than 50% biofilm inhibition at the concentrations of 468–3750.0 µg/mL and 234.4–1875 µg/mL for ATCC 19606 and ATCC BAA-3252, respectively. On the other hand, there was no biofilm formation inhibition > 50% by the plant extract at the concentrations of below 58.6 µg/mL and above 7500 µg/mL (Table 3). Some differences between strains were statistically significant (Figure 3).

The effect of ethanol on the biofilm formation by bacteria was also evaluated for both strains, with the ethanol diluted in a *v/v* ratio analogous to that of the extract. The MBIC values obtained were two to four times higher (3750–7500 μg/mL) than those obtained for the untreated strains. The MBIC/MIC ratio indicates that both the alcohol extract and ethanol inhibited biofilm formation, particularly concerning *A. baumannii* ATCC BAA-3252 cells. Statistically significant differences were observed between cells treated with the extract or ethanol at most concentrations (Table 3) for the ATCC 19606 strain, particularly at a subinhibitory concentration of ¼ MIC (117.2 μg/mL; *p* = 0.0001). Similar observations were noted for the ATCC BAA-3252 strain at a concentration of ½ MIC (117.2 μg/mL; *p* = 0.0017).

### 3.4. Biofilm-Related Genes

The presence of biofilm-related genes was determined via PCR with specific primer pairs followed by agar gel electrophoresis. Both reference strains of *A. baumannii* presented the same set of genes, except for c*suA*, which was present only in the *A. baumannii* ATCC 19606 strain.

### 3.5. Effects of Sub-MICs on Biofilm Formation Gene Expression

**Figure 4:**
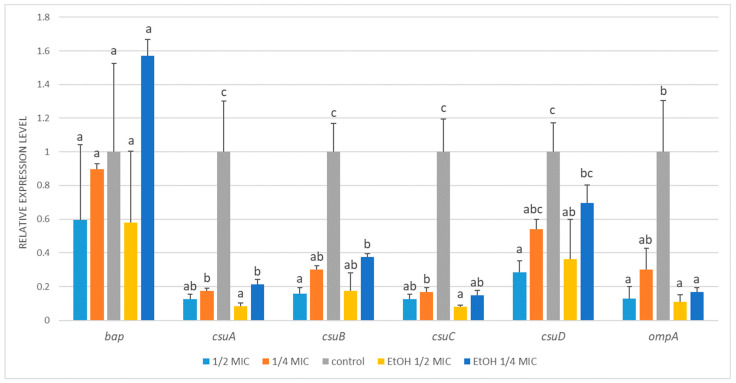
Relative expression of target genes in *Acinetobacter baumannii* ATCC 19606 subjected to ½ MIC (½ MIC of alcoholic *A. montana* flower extract); ¼ MIC (¼ MIC of alcoholic *A. montana* flower extract), EtOH ½ MIC (½ MIC of ethanol), EtOH ¼ MIC (¼ MIC of ethanol). The data represent the means ± SDs (*n* = 3). Means concerning the same gene, followed by different letters, are significantly different according to Tukey’s test at *p* < 0.05.

**Figure A2:**
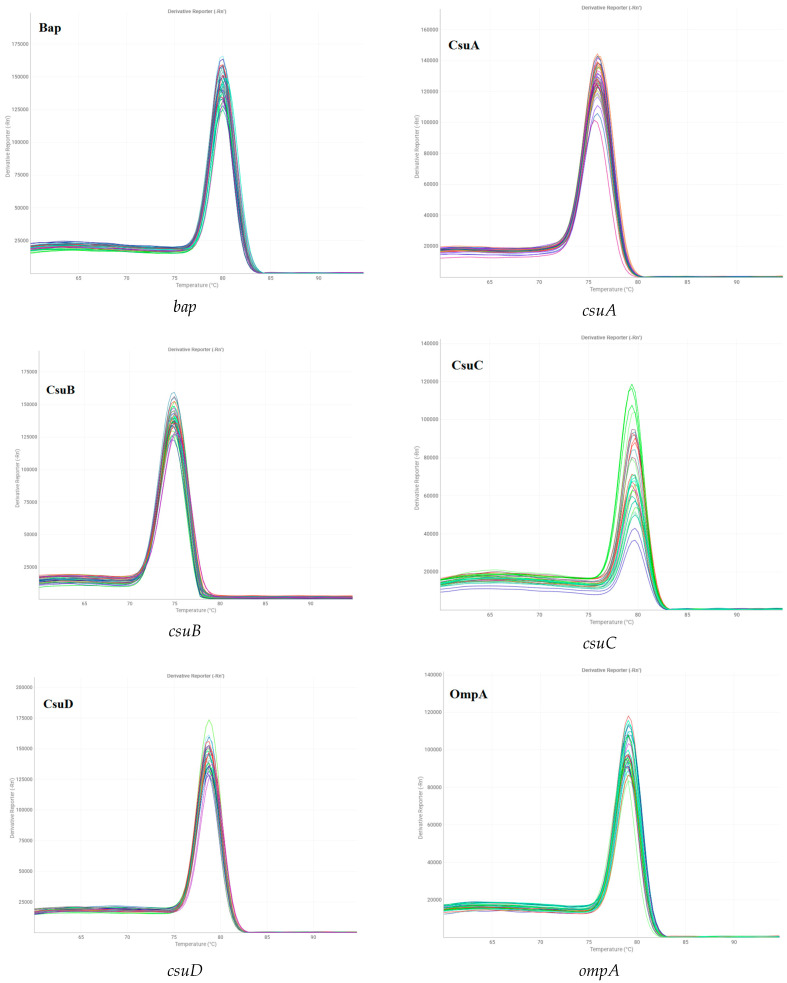

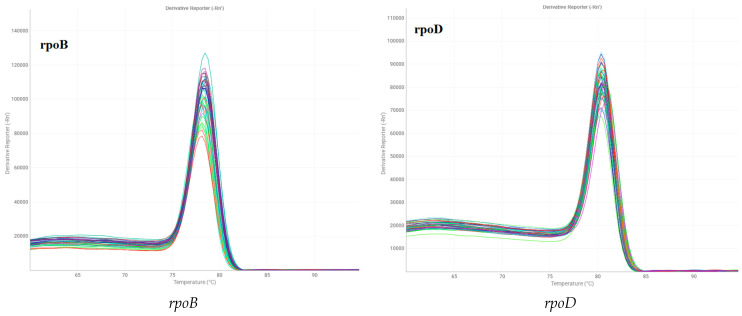
Melting-curve analysis of all selected biofilm-related genes in *Acinetobacter baumannii* ATCC 19606.

*A. baumannii* ATCC 19606 exhibited significant transcriptional alterations in all tested biofilm-related genes, except for *bap*, whose expression remained unchanged regardless of the treatment (Figure 4). The expression of the c*suA-C* genes was markedly downregulated following treatment with either arnica extract or ethanol (Figure A2). For example, compared with the control, exposure to sublethal ½ MIC of the *A. montana* flower extract decreased *csuA-C* transcript levels by approximately 6- to 8-fold. Likewise, ¼ MIC of the extract caused a 3- to 6-fold decrease in their transcription. The changes induced by ethanol treatment were analogous and showed no statistically significant difference compared to those caused by the extract. The decrease observed in *csuD* expression was less pronounced and reached statistical significance compared to the control only at ½ MIC of both the extract and ethanol treatments. In the case of *ompA*, its expression decreased significantly in response to the ½ MIC of the extract and ethanol (by 8- and 9-fold), while the reduction at the ¼ MIC of the extract did not reach statistical significance.

**Figure 5:**
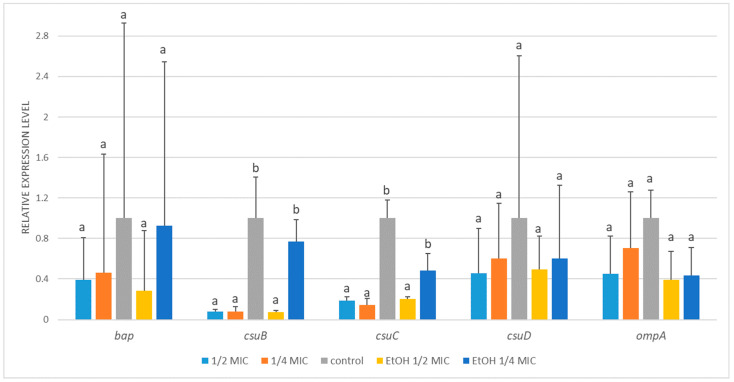
Relative expression of target genes in *Acinetobacter baumannii* ATCC BAA-3252 subjected to ½ MIC (½ MIC of alcoholic *A. montana* flower extract), ¼ MIC (¼ MIC of alcoholic *A. montana* flower extract), EtOH ½ MIC (½ MIC of ethanol), EtOH ¼ MIC (¼ MIC of ethanol). The data represent the means ± SDs (*n* = 3). Means concerning the same gene, followed by different letters, are significantly different according to Tukey’s test at *p* < 0.05.

While the mRNA levels of *bap*, *csuD*, and *ompA* exhibited a modest reduction in *A. baumannii* ATCC BAA-3252 following the treatments, the changes were not statistically significant (Figure 5). Exposure to ½ MIC and ¼ MIC of *A*. *montana* flower extract resulted in strong transcriptional inhibition of *csuB* (~13-fold), with ½ MIC ethanol treatment producing a similar effect. In contrast, ¼ MIC ethanol did not significantly affect *csuB* expression. A similar trend was observed for *csuC*, with mRNA levels decreasing approximately 5- to 7-fold in response to ½ MIC and ¼ MIC of extract, as well as ½ MIC ethanol. In contrast, ¼ MIC ethanol caused only a minor, nonsignificant decrease.

## 4. Discussion

*Arnica montana* L. extracts have been the subject of extensive research over the course of many years. Their antibacterial properties have been demonstrated to be particularly effective against Gram-positive bacteria, such as *Staphylococcus aureus*, including methicillin-resistant ATCC 25923 MRSA strains, as well as Gram-negative bacteria, such as *Escherichia coli* ATCC 25922, and yeasts, such as *Candida albicans* ATCC 60193 [19]. The authors demonstrated that all of the tested extracts successfully inhibited microorganisms, with *S. aureus* exhibiting the greatest sensitivity (MIC ranging from 0.10 to 0.31 mg/mL), *E. coli* exhibiting intermediate sensitivity (MIC ranging from 1.23 to 2.58 mg/mL), and *C. albicans* exhibiting the greatest resistance (MIC ranging from 1.41 to 5.16 mg/mL). These results varied depending on extraction conditions such as temperature (36–64 °C) and pressure (5.9–34.1 MPa). Kryvtsova and Koščová [22] studied ethyl and methyl extracts produced from the inflorescences of *A. montana* L. against staphylococci, including the biofilm-forming strain *S. aureus* CCM 4223. The authors concluded that the arnica extract demonstrated antimicrobial effects, including activity against clinical MRSA and biofilm-producing *S. aureus* strains. At the same time, the activities of the ethyl and methyl extracts were comparable. It was also demonstrated that introducing a 0.1% extract solution reduced biofilm formation by 92%. As the extract concentration decreased, the antibiofilm activity gradually declined. Yalgi and Bhat [20] reported good antibacterial activity of *A. montana* tincture against *S. mutans* and *E. faecalis*, with MIC values of 62.5 mg/mL and 8 mg/mL, respectively. Meanwhile, Amato et al. [21] tested the antimicrobial activity of an *A. montana* ethanolic extract against various bacteria and *Candida* spp. yeasts. They have demonstrated a growth of all bacterial strains at different extract concentrations, except for *B. subtilis* and *P. aeruginosa*. Under these experimental conditions, *A. montana* did not demonstrate any activity that would suggest its use as an antimicrobial agent. In addition, it has been demonstrated that arnica extracts possess the capacity to impede the development of bacterial biofilms [22]. This property holds significant promise for applications in the field of topical antibiotic and antifungal therapy, as well as in the management of various forms of oral and oropharyngeal inflammation. However, the available literature does not provide strong evidence that Arnica is effective against *Acinetobacter* spp. bacteria. Treatment of *Acinetobacter* infections currently focuses on novel antibiotic therapies or other methods rather than traditional remedies, such as arnica. In this study, the ethanolic extract of *A. montana* flowers exhibited strong bactericidal properties against *Acinetobacter* spp. reference strains, with MIC values ranging from 234.4 to 468.75 µg/mL, and the MBC values at the same level of 937.5 µg/mL. The MIC values showed that arnica ethanol extract was active against planktonic cells of the reference strains of the species *A. baumannii*. We have observed a decreased bacterial growth when the concentration of the ethanolic extract exceeded 468.8 µg/mL. For *Acinetobacter* ATCC 19606, planktonic cell growth was inhibited by 81.3–94.3%, while for ATCC 3252, inhibition ranged from 88.4–95.0%. The presence of ethanol inhibited the growth of planktonic cells of these bacteria (at concentrations corresponding to MIC of >468.8 μg/mL of the extract), with growth inhibition ranging from 91.5 to 96.3% for *A. baumannii* ATCC 19606 and from 95.6% to 96.3% for *A. baumannii* ATCC 3252. The extract was 2-fold as effective at inhibiting bacterial growth as pure ethanol at a lower concentration. This suggests that the arnica extract could work by combining or strengthening the effects of the solvent and the substances it contains. At high concentrations, the solvent itself was more toxic to bacteria than the extract containing plant bioactive compounds (e.g., STLs or flavonoids). Moreover, subinhibitory concentrations of the extract inhibited biofilm formation by *A. baumannii*, likely affecting processes such as surface colonization, adhesion, and biofilm development. As demonstrated in the extant literature, subinhibitory concentrations of plant extracts have the capacity to inhibit biofilm formation in many bacterial strains, including Gram-negative rods. Several studies have reported that certain plant extracts, such as *Piper betle* [41], have the capacity to substantially reduce the growth of biofilms and even eradicate mature biofilms at concentrations of **¼** MIC or ½ MIC. This activity is attributed to the extract’s ability to modify bacterial surface proteins, disrupt the formation of the biofilm polysaccharide matrix, and potentially interfere with *quorum-sensing* mechanisms. Furthermore, several other mechanisms of action have been postulated. The presence of plant extracts has been demonstrated to lead to the formation of an unfavorable layer on biotic and abiotic surfaces, thereby preventing bacteria from adhering to and colonizing new surfaces. Medicinal plant extracts rich in essential oils (e.g., *Thymus zygis*, *Rosmarinus officinalis*, *Origanum majorana,* and *Lippia graveolens*) have the capacity to penetrate and destabilize the intricate polysaccharide matrix that serves as a structural basis for bacterial biofilms, thereby inducing their detachment [42,43]. Moreover, these extracts disrupt communication signals via *quorum sensing* employed by bacteria to orchestrate the formation of robust and resilient bacterial biofilms, thereby impeding the progression of mature biofilms [10,42,43].

Biofilm formation plays a critical role in the emergence and persistence of multidrug-resistant *Acinetobacter* infections, as it protects bacteria from environmental stressors and antimicrobial agents, thereby facilitating their prolonged presence in clinical settings [25,26,44]. Key *A. baumannii* genes involved in biofilm formation include *bap*, which encodes a biofilm-associated protein; the *csu* operon, which is responsible for building pili for attachment; and genes such as *ompA*, *pgaA*/*pgaB*, *epsA*, *bfmR*-*bfmS*, and *ptk*, which contribute to biofilm structure, maturation, and interaction with surfaces [45,46,47,48,49,50]. These genes are often associated with antibiotic resistance and the persistence of *A. baumannii* in hospital environments, making them important targets for understanding and combating infection.

Bap (biofilm-associated protein) is a large protein expressed on the bacterial cell surface. It is assumed to be crucial for the intercellular adhesion process, accumulation and aggregation of bacterial cells, biofilm development, and the formation of complex, three-dimensional structures [44]. Many *A. baumannii* isolates carrying the *bap* gene form biofilms on both biotic and abiotic surfaces under stationary conditions [51]. The essential role of the *bap* gene in biofilm formation was confirmed in *Staphylococcus xylosus*, where all biofilm-producing strains contained *bap*, whereas the only biofilm-negative strain, TMW 2.1602, carried a truncated *bap* sequence [45].

The *csu* operon, comprising six genes (*csuA/B*, *csuA*, *csuB*, *csuC*, *csuD*, and *csuE*), is conserved in most clinically significant *Acinetobacter* spp. species and encodes a chaperone-usher pili assembly system important for surface adherence and biofilm formation [52]. An early study by Tomaras et al. [53] demonstrated that mutation of *csuC* or *csuE* in *A. baumannii* ATCC 19606 led to the loss of pili observed in the parental strain and biofilm deficiency. Another study reported that mutants of *A. baumannii* ATCC 17978 lacking *csuD* also did not produce Csu pili and were completely incapable of forming visible biofilm structures [52]. Findings from a different study revealed that major epidemic *A. baumannii* ST191 clones presented significantly greater biofilm masses than minor epidemic and sporadic clones did. This enhanced biofilm formation correlated with increased expression levels of the *csuC*, *csuD*, and *csuE* genes in major epidemic clones. Although *bap* expression was elevated in both major and minor epidemic clones relative to sporadic clones, it did not differ significantly between the two epidemic groups. These results suggest that the differential expression of specific *csu* operon genes plays a key role in biofilm formation variability among epidemic clones [54]. The carbapenem-resistant *A. baumannii* mutant 37662RM2, which lacks capsule synthesis but exhibits markedly enhanced biofilm formation, showed significant overexpression of the *csu* operon and its regulatory genes *bfmR-bfmS* compared to the wild-type 37662 strain. In contrast, the expression of *bap* was only marginally altered. These data imply that the increased biofilm phenotype in 37662RM2 is driven primarily by the upregulation of the *csu* operon and its regulators, highlighting their critical role in biofilm development independent of capsule production [55]. Ahmad et al. [47] highlighted the multifaceted role of Csu pili in *A. baumannii* pathogenicity. The Csu pili were shown to facilitate bacterial adherence to lung epithelial cells and contribute to the formation of structurally complex biofilms. Kishii et al. [56] investigated the relationship between biofilm formation and the presence and transcriptional activity of biofilm-associated genes in clinical *A. baumannii* strains collected from hospitals in Japan. They found that 73.7% of the isolates carried the complete *csu* operon, whereas the remainder lacked these genes. In contrast, *ompA* and *abaI* (*quorum-sensing* autoinducer) were present in all the isolates. Strains lacking the *csu* operon exhibited weak biofilm formation, whereas *csu*-positive strains expressing the operon genes showed strong biofilm production. Interestingly, some *csu*-positive strains did not express the operon, yet formed intermediate biofilms. This suggests that pili may not be strictly required for biofilm development and that other adhesion factors may compensate for the absence of the functional Csu system. OmpA (outer membrane protein A) is widely recognized for its role in bacterial adhesion and biofilm formation, contributing to the overall stability of the biofilm structure. Moreover, the role of *ompA* in the multidrug resistance phenotype of *A. baumannii* has been demonstrated, as disruption of the *ompA* gene resulted in decreased MICs for chloramphenicol, aztreonam, and nalidixic acid [50]. In another study [57], a Δ*ompA* mutant of *A. nosocomialis* ATCC 17903 exhibited significantly reduced biofilm formation and decreased adherence to human epithelial cells. Deletion of the *ompA* gene significantly reduced bacterial dissemination between organs and impaired the development of secondary pneumonia in a murine infection model [58]. A separate study demonstrated a link between elevated *ompA* expression and the adaptive response of *A. baumannii* persister cells to meropenem exposure, indicating that upregulation may enhance bacterial survival during antibiotic treatment and contribute to persistence mechanisms [59]. Furthermore, strains with elevated protein expression were more invasive [60]. Na et al. [61] screened small molecules targeting *ompA* promoter activity in *A. baumannii*. The study identified three chemical compounds that inhibited expression in the *A. baumannii.* Encinales et al. [58] proposed that blocking OmpA could serve as a novel therapeutic strategy against Gram-negative bacilli infections, particularly those caused by *A. baumannii*. These findings highlighted that overexpression of the OmpA protein significantly correlates with higher risks of pneumonia, bacteremia, and increased mortality, underscoring the pivotal role of the OmpA protein in disease severity and clinical outcomes.

Taken together, the genes listed above contribute to *A. baumannii*’s ability to form biofilms, which protect the bacteria from host immune responses and antibiotics, leading to chronic and difficult-to-treat infections. In a study by Kasperski et al. [62], 72% of 100 tested *A. baumannii* isolates obtained from hospitalized patients were strong biofilm producers and consistently harbored the *bap*, *csuE,* and o*mpA* genes. Another study involving 107 *A. baumannii* isolates from both clinical and environmental sources reported that 31.2% of clinical strains and 58.7% of environmental strains were strong biofilm producers, with *ompA* detected in all isolates and *bap* present in 89 strains [50]. Saadati et al. [48] reported a high prevalence of biofilm-associated genes in 100 clinical *A. baumannii* isolates, with all strains positive for *ompA* and 89 harboring the *bap* gene. Additionally, all the isolates were MDR, and 79% were extensively drug resistant (XDR). Yang et al. [44] found that 144 of 154 *A. baumannii* strains produced biofilms, with 79.2% positive for *bap*, 68.8% for *csuE*, and 91.6% for *ompA*. The study demonstrated that strains harboring *bap*, *ompA*, and *csuE* genes formed stronger biofilms compared to those lacking these genes. Interestingly, all ten *A. baumannii* nonbiofilm-forming strains contained the *ompA* gene, whereas *bap* and *csuE* were detected in six and seven of these strains, respectively. Amin et al. [63] analysed 64 *A. baumannii* isolates obtained from burn infection cases, revealing important insights into the presence and expression of biofilm-associated genes. While genes such as *ompA* and *abaI* were detected in both biofilm-producing and nonproducing isolates, the *bap* gene was exclusively found among biofilm producers. Furthermore, expression levels of all biofilm-related genes were significantly higher in strong biofilm producers compared to moderate or weak producers. These findings indicate that both the presence and expression levels of biofilm-associated genes crucially impact the bacteria’s ability to form biofilms.

In this study, in addition to the assessment of the antibacterial and antibiofilm activities of the *A. montana* ethanolic extract, its influence on the expression of biofilm-related genes in *A. baumannii* was studied. Transcriptional regulation of biofilm-associated genes (*bap*, c*suABCD*, *ompA*) was evaluated following exposure to subinhibitory ½ MIC and ¼ MIC of *A. montana* extract, as well as in solvent controls at corresponding concentrations, and compared with those of untreated bacteria. In most cases, the treatment resulted in pronounced downregulation of the target genes, except *bap*, whose expression remained unaffected.

In contrast to our findings, the methanolic extract of *Nothoscordum bivalve*, which inhibited biofilm formation in *A. baumannii*, slightly upregulated *bap* expression at 50% MBC = 3.74 mg/mL and 75% MBC = 5.61 mg/mL. Yet, bacteria treated only with solvent (methanol at 50 and 75% of the MBC) showed no changes in *bap* expression, which is congruent with our results [64]. A related study on *A. baumannii* examined the impact of nanoparticles on biofilm formation and the expression of the *bap* and *csu* genes. Compared with the control, treatment with silver nanoparticles at the MIC (6.25 μg/mL) led to reduced expression of *bap*, *csuC*, and *csuE*. In contrast, gold nanoparticles conjugated with vancomycin at MIC (0.625 μg/mL) upregulated *csuC* and *csuE* but downregulated *bap* relative to controls. Despite these differing transcriptional responses, both nanoparticle types demonstrated inhibitory effects on biofilm formation, suggesting distinct underlying mechanisms of action [65].

Various plant extracts and compounds vary in their inhibition of bacterial biofilm formation, underscoring the complexity and specificity of their antibiofilm actions. Choudhary et al. [66] demonstrated that eugenol and geraniol exhibit antibiofilm properties in *A. baumannii*, concurrently leading to a downregulation of *csuE* and inhibition of biofilm formation by interfering with pilus assembly mechanisms. A recent investigation of the *Origanum vulgare* ethanolic extract demonstrated its impact on virulence-associated genes in dental plaque bacteria. In *Streptococcus mitis* SA5, *bapA1* expression was significantly decreased at the highest extract concentration (0.781 mg/mL), with no significant effect at lower doses [67]. In *S. epidermidis*, propolis interfered with biofilm development by reducing the transcription of biofilm-associated genes [68]. Similarly, *Cleome droserifolia* extract was found to repress transcription of biofilm-related genes in 43% of the tested *S. aureus* isolates, providing a molecular basis for its antibiofilm activity [69]. In the biofilm-forming *Proteus mirabilis* isolates, an extract of the traditional medicinal plant *Alhagi maurorum* was shown to decrease biofilm production while displaying concentration-dependent antiadhesive and antiquorum-sensing activities. These findings were supported by transcriptomic evidence indicating the downregulation of genes associated with biofilm regulation [70]. The exposure of *S. aureus* to 0.5 × MIC *Thesium chinense* ethyl acetate extract led to transcriptional reprogramming, with 531 genes upregulated and 340 genes downregulated. Notably, the suppressed genes encompassed those involved in biofilm formation [71]. Ranfaing et al. [72] investigated the transcriptomic effects of cranberry proanthocyanidins (190 µg/mL), propolis (102.4 µg/mL), and their combination on a clinical uropathogenic *Escherichia coli* strain via microarray analysis. Propolis potentiated cranberry proanthocyanidins’ effects, leading to downregulation of more adhesion- and biofilm-related genes. In another study, Liu et al. [73] demonstrated that the methanol-phase extract of *Rumex madaio* exerts species- and strain-specific transcriptional changes. Specifically, *Vibrio alginolyticus* ATCC 17749 upregulated *quorum-sensing*-related genes, whereas *Vibrio parahaemolyticus* B4-10 predominantly downregulated their transcription. In contrast, *quorum sensing* was not among the significantly affected metabolic pathways in *V. parahaemolyticus* ATCC 17802, highlighting strain-dependent transcriptional variability. These findings underscore the complexity of plant extract–bacteria interactions, where conserved virulence traits such as *QS* may be targeted in various ways depending on the species and strains, ultimately influencing the ability of these pathogens to initiate and sustain biofilm formation.

This is congruent with our findings, which also indicate strain-dependent responses to plant-derived antimicrobials. Our study utilized two *A. baumannii* strains: ATCC 19606, a well-established reference strain commonly employed in biofilm and virulence research due to its relative antibiotic susceptibility, and ATCC BAA-3252, a clinically derived multidrug-resistant isolate that reflects the therapeutic challenges posed by contemporary clinical infections. While sub-MIC concentrations of *A. montana* extract significantly downregulated *csuD* and *ompA* in *A. baumannii* ATCC 19606, no comparable transcriptional changes were detected in ATCC BAA-3252. This variability underscores the heterogeneity of virulence regulation among different isolates and highlights the importance of evaluating plant-based antibiofilm agents across multiple strains.

These results are in line with the observations of Khosravy et al. [49], who analyzed extensively drug-resistant (XDR) *A. baumannii* strains isolated from intensive care patients. They found that clinical isolates showed upregulation of biofilm genes compared to the reference strain ATCC 19606, including approximately 12-fold higher *bap* expression. Kakavan et al. [46] also reported a significant association between *bap* overexpression and antibiotic resistance in a study of 100 clinical *A. baumannii* isolates obtained from hospitalized patients. Their findings demonstrated a direct correlation between biofilm production capacity and *bap* expression, with strong biofilm-producing isolates exhibiting the highest *bap* expression levels. These observations suggest that the *bap* gene may represent a promising target for the development of novel therapeutic strategies against *A. baumannii*. Although the *bap* gene plays a critical role in biofilm formation, our findings indicate that the antibiofilm activity of *A. montana* extract may not be directly mediated through transcriptional regulation of this gene, as its expression remained unchanged during treatment. This implies the extract inhibits biofilm development by targeting other virulence factors linked to biofilm formation. According to the obtained results, *A. baumannii* ATCC 19606 exhibited downregulation of the *csuABCD* operon as well as the *ompA* gene, indicating a broader repression of genes related to biofilm formation and outer membrane structure. In contrast, strain ATCC BAA-3252 displayed downregulation limited to *csuB* and *csuC*, suggesting a more selective impact on biofilm-associated genes. This differential gene expression implies that ATCC 19606 may undergo a more comprehensive alteration in biofilm assembly and cell surface properties compared to ATCC BAA-3252. Furthermore, *A. baumannii* ATCC 19606 and ATCC BAA-3252 exhibited similarities and differences in their antimicrobial susceptibility profiles. Both strains shared the same MBC of 937.5 µg/mL, indicating a comparable ethanolic extract concentration required to kill the planktonic cells of bacteria. However, MIC values differed: ATCC 19606’s MIC (468.75 µg/mL) was twice that of ATCC BAA-3252 (234.4 µg/mL). This suggests that while both strains require the same bactericidal level of the arnica ethanolic extract, ATCC BAA-3252 is inhibited at a lower concentration, indicating slightly higher susceptibility. This difference in MIC values highlights strain-specific variation in antimicrobial efficacy, despite similar bactericidal thresholds. A comprehensive understanding of these differences is crucial for the development and optimization of targeted therapeutic strategies against *A. baumannii* and requires further detailed investigations.

In the available literature, studies explicitly comparing the effects of plant extracts and corresponding solvent controls on bacterial gene expression are scarce. Our study uniquely addresses this gap by including ethanol solvent controls matched to the extract concentrations. Antibacterial activity assay demonstrated that for both *A. baumannii* strains tested, the MIC for the solvent control was two times higher compared to the MIC of the Arnica extract. These results clearly highlight that the inhibitory effects observed are predominantly mediated by bioactive compounds present in the extract rather than the ethanol alone. However, our findings also confirm that ethanol, particularly at elevated concentrations, can contribute significantly to growth inhibition.

In strain ATCC 19606, the solvent control elicited gene expression changes similar to those induced by the extract, suggesting solvent-driven transcriptional influence. Conversely, in strain ATCC BAA-3252, ethanol at ¼ MIC failed to suppress expression of *csuB* and *csuC*, whereas the Arnica ethanolic extract at equivalent concentration did. This discrepancy emphasizes that in strain ATCC BAA-3252, gene expression modulation is principally attributed to phytochemicals in the extract rather than solvent effects.

These observations highlight a limitation of using ethanol as a solvent, as its impact may vary with strain sensitivity and can mask or mimic gene regulatory effects. Possible explanations for the different responses include strain-specific ethanol tolerance or adaptation, as well as the presence of synergistic or antagonistic phytochemicals in the extract influencing transcriptional regulation. The limitations of this study include possible confounding effects of the solvent on gene regulation and the need to isolate active compounds to clarify their precise roles. Further research using solvent-free formulations or purified compounds is needed to elucidate the mechanisms underlying these observations. Nevertheless, our findings emphasize that the inhibitory effect on *csu* operon expression in ATCC BAA-3252 stems from active phytochemicals within the Arnica extract, since ethanol alone failed to elicit comparable gene suppression. This highlights the contribution of extract components beyond the solvent in modulating bacterial gene expression

As stated above, most published research focuses on the effects of extracts themselves, often overlooking or inadequately controlling for solvent influences. Our study uniquely contributes by including an ethanol solvent control at concentrations equivalent to those in the extract treatments. This approach enables a clearer differentiation between gene expression changes caused by bioactive compounds and those potentially induced by the solvent. The findings underscore the importance of incorporating such controls to accurately assess extract-specific transcriptional effects and avoid misinterpretations arising from solvent artifacts. Incorporation of solvent controls thus represents a critical methodological consideration for future investigations aiming to elucidate extract-mediated gene expression modulation.

## 5. Conclusions

The ethanolic extract of *A. montana* L. flowers contains bioactive compounds that exhibit bactericidal effects against *A. baumannii* and inhibit its biofilm formation. This bactericidal activity, combined with the downregulation of key biofilm-associated genes, specifically the *csu* operon and *ompA*, underscores the extract’s dual mode of action in targeting both bacterial viability and biofilm development. By disrupting pilus assembly and bacterial adhesion mechanisms, the extract impairs biofilm formation and increases bacterial susceptibility to antimicrobial agents. These findings highlight the potential of ethanolic *A. montana* L. extracts as a complementary therapeutic strategy against infections caused by antibiotic-resistant *A. baumannii* strains. However, further studies are required to comprehensively elucidate the antimicrobial mechanisms involved.

## Figures and Tables

**Table 1:** Primer sequences and characteristics.

Gene Symbol	Accession	Primer Sequence 5′ → 3′	Primer Concentration	Amplicon Size	R^2^	Efficiency
*bap*	KT717635	F: GAGGGAACTTCTGCAAAACTTTC	400 nM	108 bp	0.999	97.10%
		R: CAGACGTATGACTGCATTGGT				
*csuA*	AY241696.1	F: TTTTGGTGAAGCTACCACAGC	400 nM	91 bp	0.998	99.72%
		R: ACCAGCACACTCGATCTGAAA				
*csuB*	AY241696.1	F: CACCTACAAACCGTCTGGCA	400 nM	78 bp	0.998	106.23%
		R: CGGCCGTATGGGGTTCATTA				
*csuC*	AY241696.1	F: CCGACTTTCTGATGGCAACG	100 nM	198 bp	0.996	90.97%
		R: AGCTCAGACTGGCCTTGTTG				
*csuD*	AY241696.1	F: TGAAGGTAACGCCGAGTCTG	400 nM	145 bp	0.996	108.92%
		R: TCGAACAGAACTTCCCCACG				
*ompA*	OL347635.1	F: CGACAGCAAAATCAAGCCGT	100 nM	132 bp	0.999	92.41%
		R: CAGAAAGCACCAACACCAGC				
*rpoD*	CP045110.1	F: GTTGCTGAAGAAGAAGCTGCTG	100 nM	101 bp	0.997	85.85%
		R: ACTGTACCCATTTCACGCATGTA				
*rpoB*	CP045110.1	F: ACGCCTAAAGGTGAAACTCAGTTAA	100 nM	110 bp	0.995	96.21%
		R: GTACCAGATGGAACACGTAAAGATG				

**Table 2:** Results of the quantitative antibacterial assay against *Acinetobacter baumannii* planktonic cells.

Concentrations [µg/mL]	*A. baumannii* ATCC 19606			*A. baumannii* ATCC BAA-3252		
Type of Solution	AqE	EtE	EtOH	AqE	EtE	EtOH
MIC	>30,000	468.75	937.5	>30,000	234.4	468.75
MBC	n.d.	937.5	937.5	n.d.	937.5	1875
MBC/MIC ratio	n.d.	2	1	n.d.	4	4
type of activity (+) bactericidal (−) bacteriostatic	n.d.	(+)	(+)	n.d.	(+)	(+)

Explanatory notes: AqE—aqueous extract; EtE—ethanolic extract; EtOH—ethanol (a series of double ethanol dilutions ranging from 0.03 to 70.0%, corresponding to dilutions of the tested extracts); n.d.—not defined; the MIC values presented are the average of at least three replicates; no standard deviation was observed, and all results equaled their average.

**Table 3:** The effect of arnica alcohol extract on the growth of *Acinetobacter baumannii* biofilm-forming cells determined based on minimal biofilm inhibitory concentration (MBIC) values and biofilm inhibition.

Strain	ATCC 19606				ATCC BAA-3252			
Treatment	EtE		EtOH		EtE		EtOH	
MBIC	1875		3750		937.5		7500	
MBIC/MIC ratio	8		4		2		16	
**Conc.** [µg/mL]	biofilm inhibition (%)	±SD	biofilm inhibition (%)	±SD	biofilm inhibition (%)	±SD	biofilm inhibition (%)	±SD
**14.6**	−87.9 **	0.256	−89.8	0.076	−191.8 **	0.121	−155.9	0.121
**29.3**	−40.8	0.064	−59.2	0.091	−73.1 **	0.161	−16.2	0.095
**58.6**	−45.4	0.060	−87.5	0.060	−50.9	0.195	−83.3	0.206
**117.2**	14.2 ***	0.081	−52.0	0.047	34.6 **	0.071	−14.5	0.056
**234.4**	39.5 **	0.049	−69.1	0.155	73.9	0.043	−34.3	0.038
**468.7**	74.9 *	0.063	2.2	0.160	77.8	0.026	−32.5	0.102
**937.5**	85.0 **	0.032	−29.4	0.113	90.3	0.008	−0.7	0.120
**1875**	84.1 *	0.053	−55.5	0.204	67.4	0.045	−51.0	0.085
**3750**	57.7 ****	0.005	71.8	0.039	−26.2 ****	0.061	20.4	0.035
**7500**	−40.6 ***	0.016	76.2	0.029	−77.7	0.097	81.3	0.016
**15,000**	−87.8 ****	0.158	83.9	0.025	−180.6	0.084	82.5	0.017
**30,000**	−89.7 **	0.050	51.5	0.054	−118.8	0.199	63.7	0.014

Explanatory notes: AqE—aqueous extract; EtE—ethanolic extract; EtOH—ethanol (concentration *v/v* in the medium corresponding to a series of dilutions); SD—standard deviation calculated from four replicates; biofilm inhibition rated on a scale of 0–100%; values < 0% indicated bacterial biofilm growth enhancement; values of 0–50% indicated weak anti-biofilm activity; values > 50% indicated good biofilm inhibition. Low inhibition values for biofilm cell growth are generally caused by the presence of insoluble solid particles and residues from the extract. High concentrations of these particles may increase the available surface area for colonization. Statistical data were analyzed using the Mann–Whitney U test. The asterisks mean statistically significant differences among one bacterial strain between extract-treated biofilm cells and ethanol-treated biofilm cells as follows: (*) are statistically significant at *p* < 0.05; (**) are significant at *p* < 0.01; (***) are significant at *p* < 0.001; (****) are significant at *p* < 0.0001. Data were based on four replicates.

## Data Availability

The raw data supporting the conclusions of this article will be made available by the authors on request.

## Abbreviations

The following abbreviations are used in this manuscript:

MDR	Multi-drug resistant pathogen
MIC	Minimum inhibitory concentration
MBC	Minimum bactericidal concentration
XDR	Extensively drug-resistant pathogen
QS	*Quorum-sensing*
DEGs	Differentially expressed genes
CRAB	carbapenem-resistant *Acinetobacter baumannii*

## Appendix A

**Table A1:** Reduction in planktonic cells growth of *Acinetobacter baumannii* reference strains treated with different concentrations of the *Arnica montana* L. ethanolic extract for 24 h.

Type of Solution		Conc. [µg/mL]	Ethanolic Extract-Treated Cells			Non-Treated Cells		Ethanol-Treated Cells		
	Parameters		Mean OD_600_	±SD	Growth Inhibition (%)	Mean OD_600_	±SD	Mean OD_600_	±SD	Growth Inhibition (%)
Strain										
ATCC 19606		14.6	1.032	0.0402	−0.3	1.010	0.002	1.0838	0.0117	−7.4
		29.3	0.9971	0.0530	1.3	1.213	0.002	1.0504	0.0352	−5.6
		58.6	0.9618	0.0638	5.4	1.141	0.001	1.0028	0.0311	1.3
		117.2	1.0089	0.0411	−1.3	1.157	0.003	0.8475	0.1010	12.1
		234.4	0.6624	0.0855	34.3	1.017	0.007	0.3696	0.0881	58.9
		468.8	0.1043	0.0119	89.4	1.008	0.001	0.0859	0.0122	91.8
		937.5	0.0604	0.0057	94.5	1.028	0.001	0.0448	0.0009	95.5
		1875	0.0784	0.0067	92.7	1.018	0.001	0.0443	0.0007	95.6
		3750	0.122	0.0049	88.1	1.032	0.005	0.0444	0.0017	95.6
		7500	0.191	0.0404	80.0	1.023	0.009	0.0429	0.0005	95.7
		15,000	0.154	0.0365	83.7	1.088	0.001	0.0421	0.0006	95.8
		30,000	0.058	0.0134	93.7	1.021	0.004	0.0382	0.0041	96.1
ATCC BAA-3252		14.6	1.019	0.0157	−7.1	1.018	0.0010	1.0601	0.0137	21.5
		29.3	1.0204	0.0333	−6.6	1.032	0.0081	1.0533	0.0216	2.6
		58.6	0.9433	0.0375	1.7	1.039	0.0006	1.0106	0.0202	21.1
		117.2	0.7346	0.1130	9.3	1.046	0.0012	0.8305	0.0416	22.3
		234.4	0.2566	0.0817	58.4	1.023	0.0010	0.1726	0.0416	79.5
		468.8	0.0738	0.0063	91.1	1.003	0.0006	0.0618	0.0046	88.1
		937.5	0.0544	0.0012	95.5	1.001	0.0010	0.0458	0.0057	90.3
		1875	0.0749	0.0019	95.6	1.007	0.0003	0.0443	0.0006	89.4
		3750	0.1213	0.0060	95.6	1.009	0.0042	0.0436	0.0005	88.5
		7500	0.1596	0.0416	95.7	1.029	0.0544	0.0431	0.0009	91.9
		15,000	0.1312	0.0189	95.8	1.024	0.0322	0.0424	0.0010	95.3
		30,000	0.0501	0.0085	96.1	1.004	0.0162	0.0373	0.0043	94.7

Explanatory notes: SD—standard deviation calculated from ten replicates; median growth inhibition (%) was reported from ten replicates for both strains in each concentration; median control of broth medium OD_570_ = 0.048 ± 0.007438638; median extract control without bacteria OD_570_ = 0.0655 ± 0.039811963.
